# Supplementary material for: Analysis of Intra-Tumoral Macrophages and T Cells in Non-Small Cell Lung Cancer (NSCLC) Indicates a Role for Immune Checkpoint and CD200-CD200R Interactions
Source: Cancers (Basel). 2021 Apr 9;13(8):1788. doi: 10.3390/cancers13081788 (PMC8069596; doi:10.3390/cancers13081788)
Supplement: Supplementary file 1 [file cancers-13-01788-s001.pdf]

cancers

MDPI

Supplementary Materials: Analysis of Intra-Tumoral Macrophages and T Cells in Non-Small Cell Lung Cancer (NSCLC) Indicates a Role for Immune Checkpoint and CD200-CD200R Interactions

Anders Tøndell, Yashwanth Subbannayya, Sissel Gyrid Freim Wahl, Arnar Flatberg, Sveinung Sørhaug, Magne Børset and Markus Haug

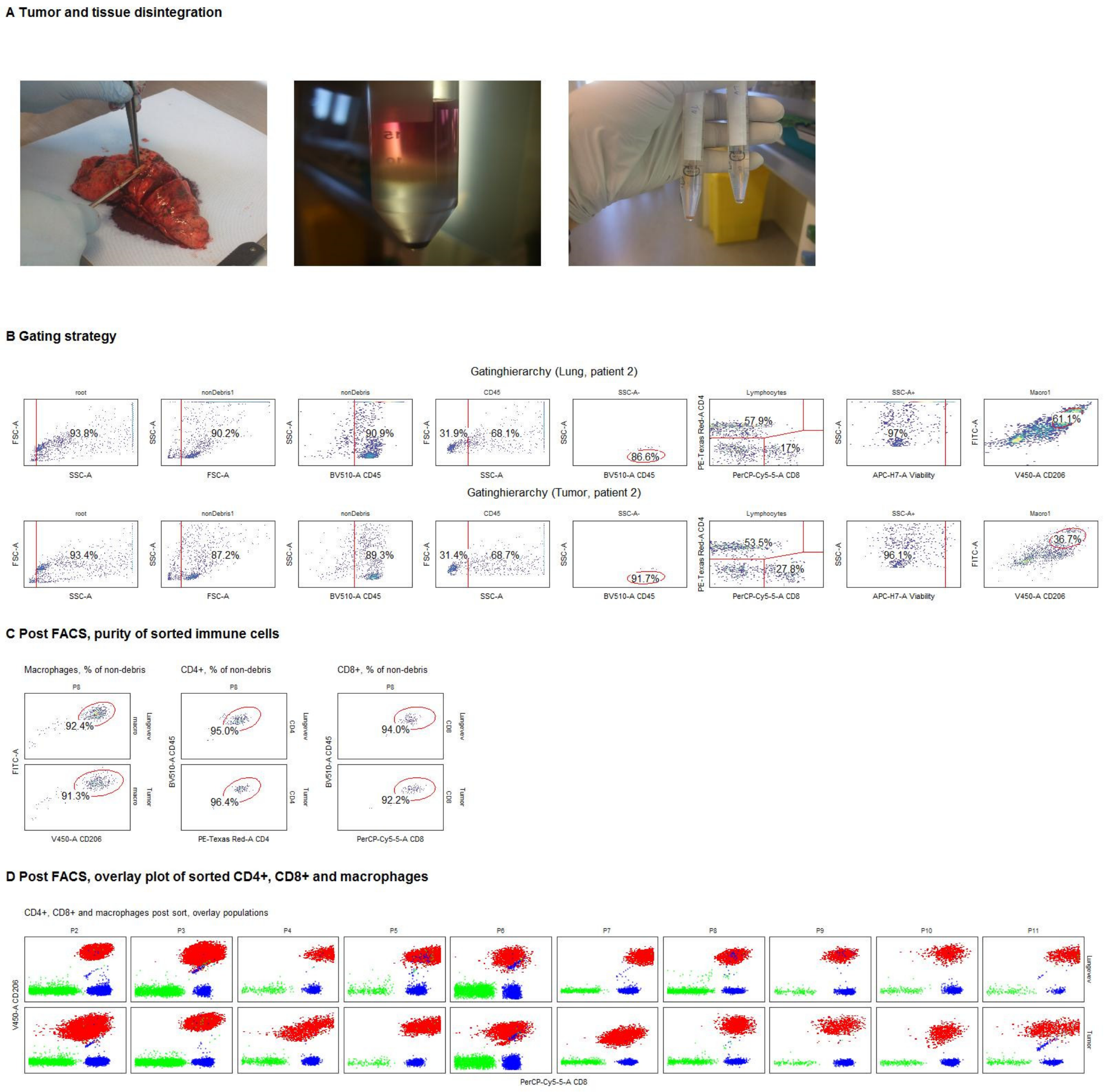

Figure S1. Isolation of immune cell subsets from tumor and lung tissue， Legend: (**A**) Immune cells from tumor and normal lung were extracted from tissue by combined enzymatic and mechanical tissue disintegration, followed by density gradient centrifugation. (**B**) Gating strategy for sorting of macrophages, CD4+, and CD8+ T cells by FACS. From the viable leucocyte (CD45^{+}) cell population, CD4+ and CD8+ T cell subsets were identified; macrophages were defined as high autofluorescence (empty FITC channel^{high}) CD45^{bright} SSc^{high} CD206^{+} cells. (**C**) Representative examples of immune cell subset purity after sorting (subcellular events excluded). (**D**) Sorted macrophage (red), CD4+ (green) and CD8+ T cell (blue) populations from patient samples displayed as overlay plots. The top row shows cells from healthy lung, while the bottom row shows the corresponding samples from tumor tissue.

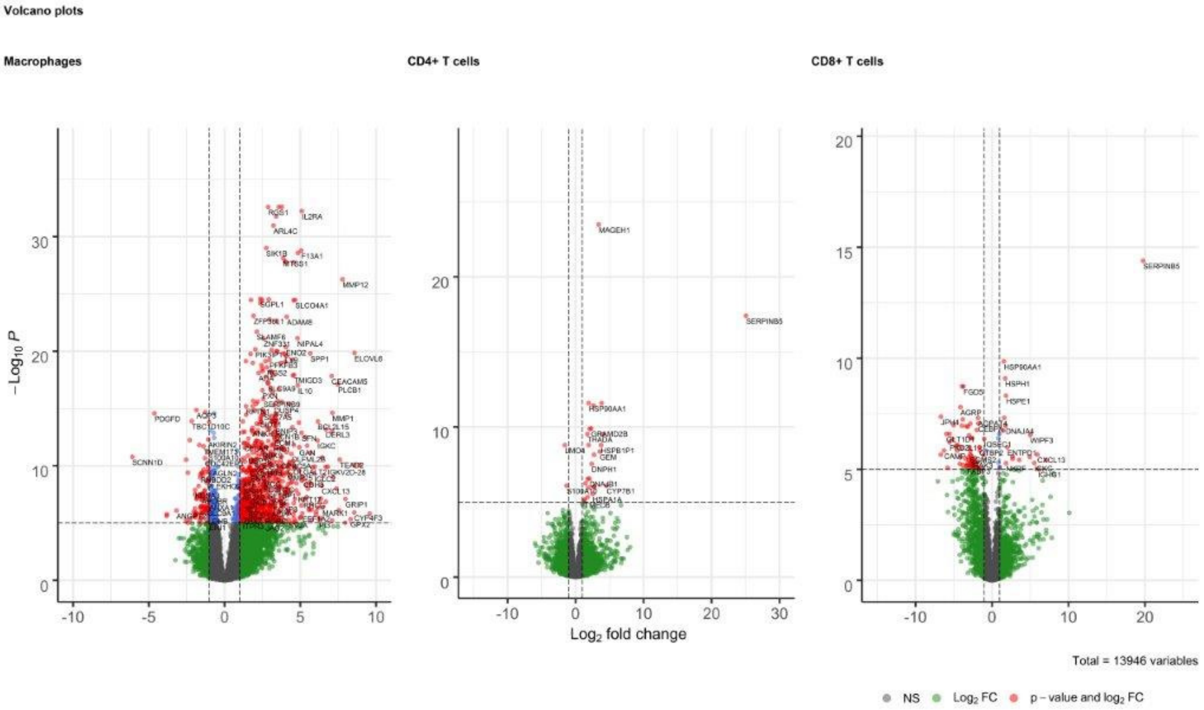

Figure S2. Volcano plots identify highly differentially expressed genes in macrophages, CD4+ and CD8+ T cells in tumor compared to lung tissue, Legend: Volcano plot displays to identify genes in immune cells that display large expression changes between tumor versus healthy lung tissue. The plots show genes in macrophages, CD4+, and CD8+ T cells separately. Gene symbols for some highly differentially expressed genes are shown.

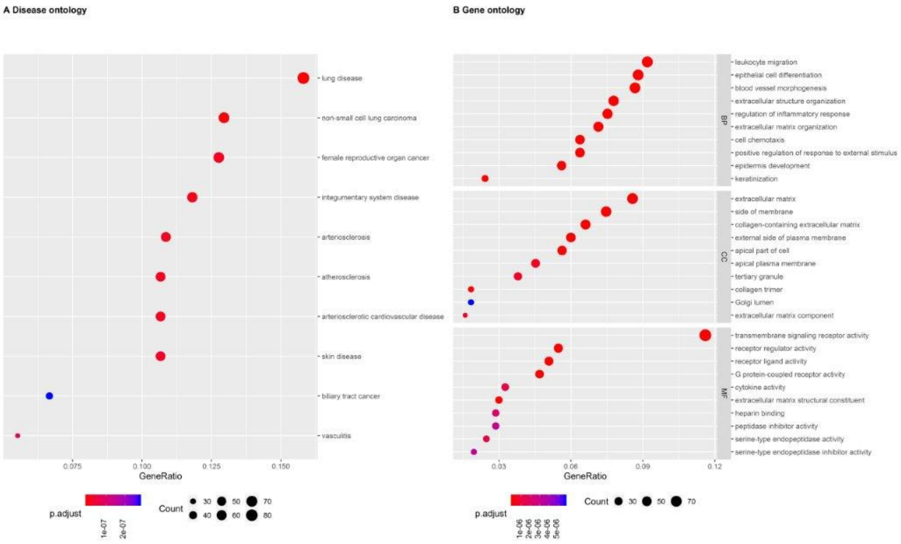

Figure S3. Disease ontology enrichment of DEGs from bulk analysis showed enrichment for the terms 'Lung disease' and 'Non-small cell lung carcinoma, Legend: (**A**) Disease ontology term enrichment analysis. Gene expression changes between tumor and non-tumor immune cells were analysed in bulk from all samples (irrespective of the cell type). The strongest DEGs enrichment was observed in the terms 'Lung disease' and 'Non-small cell lung carcinoma'. (**B**) Bulk GO term enrichment analysis. Gene expression differences between tumor and non-tumor immune cells were analysed in bulk for for GO terms belonging to biological processes (BP), cellular compartment (CC) and molecular function (MF). DEGs were most enriched in terms related to 'leukocyte migration', 'extracellular matrix' and 'Transmembrane signaling receptor activity'.

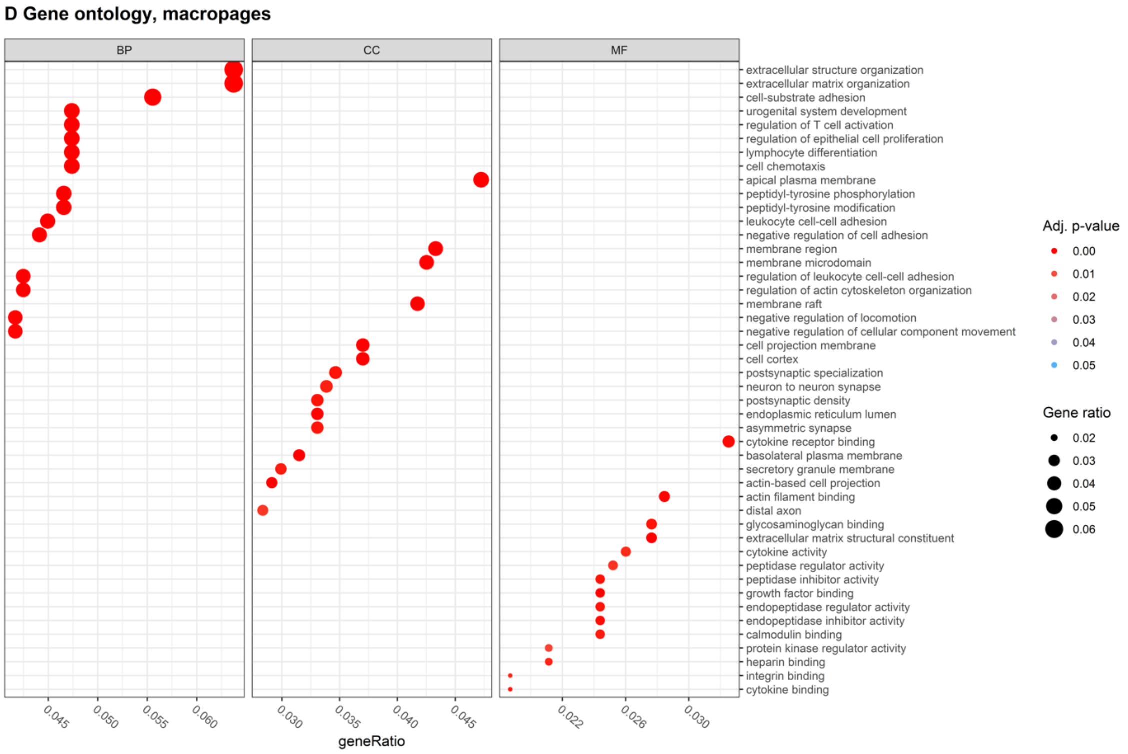

Figure S4. Gene ontology term enrichment in macrophages, *Legend*: The list of DEGs from macrophage samples (comparing gene expression in tumor and normal lung macrophages) was analyzed for gene ontology (GO) term enrichment for terms related to subontologies 'biological processes' (BP) 'molecular function' (MF), and 'cellular component' (CC). Dot color represent the adjusted p-value after overrepresentation test, dot size the ratio of differentially expressed genes (DEGs) in the macrophage samples to the number of genes in the GO term (Gene ratio).

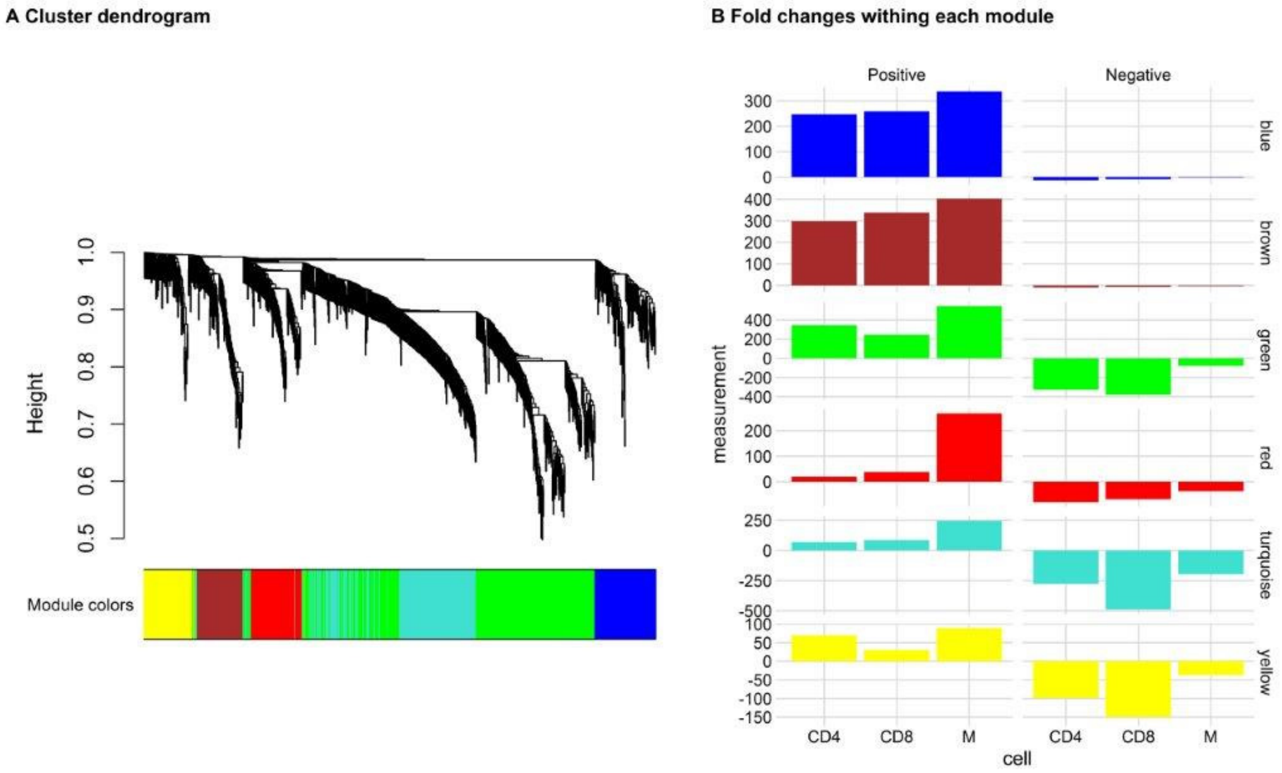

Figure S5. Network clustering analysis of transcriptome data. Legend: To identify genes with common functional origin, the pooled RNA-Seq data from macrophages (n = 11), CD4+ (n = 5) and CD8+ T cells (n = 7) in tumor and healthy tissue

was subjected to Weighted correlation network analysis (WGCNA). (**A**) Dendrogram displaying how genes with highly correlated expression patterns were grouped into 6 different modules and assigned colors. Each module color represents a cluster of genes with a possible common functional origin. (**B**) The genes in each module (colors) were grouped as either positively (left bars) or negatively regulated (right bars) in immune cells from tumor compared to those from normal lung. The bars represent the sum of the log2 fold change of the gene expression for all genes within each module for CD4+ (CD4) and CD8+ (CD8) T cells and macrophages (M) individually, for upregulated and downregulated genes respectively.

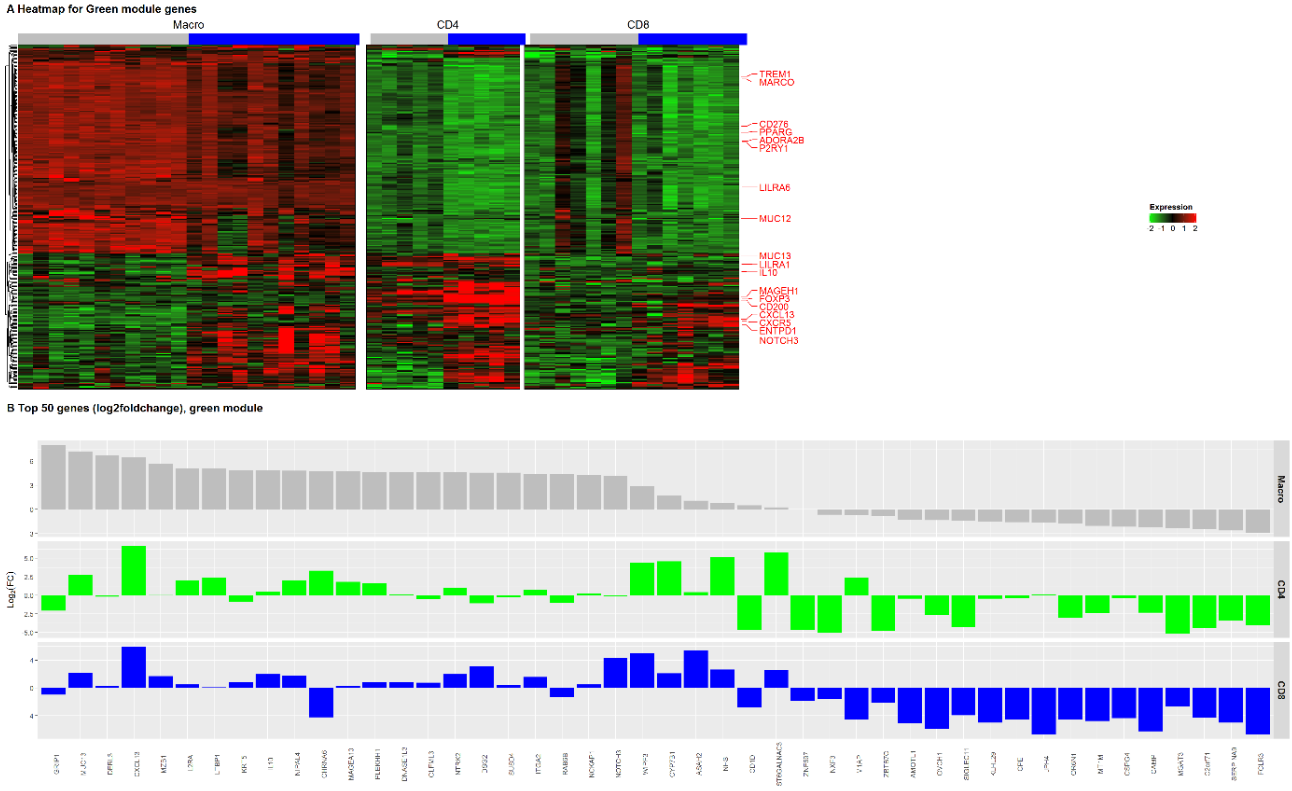

Figure S6. Differentially expressed genes between tumor and healthy tissue immune cells in a cluster of highly correlated genes identified by network clustering analysis (´green´ module), Legend: (**A**) Heatmap display of differential expression between tumor and healthy tissue for the ´green´ module genes (*n* = 319) genes in macrophages (*n* = 11), CD4+ (*n* = 5) and CD8+ (*n* = 7) T cells. The top bar denotes immune cell samples originating from healthy lung (grey) and tumor (blue). Genes with known immunosuppressive effects in the TME are highlighted, such as the adenosine receptor A2B (ADORA2B), interleukin-10 (IL10), CD39 (ENTPD1) and Forkhead box P3 (FOXP3). B) The top 50 DEGs list for the ´green´ module was generated from pooled transcriptome data from all cell types. Bar plots display expression of the individual top 50 DEGs in macrophages (grey), CD4+ (blue) and CD8+ (green) T cells (tumor versus healthy tissue). The list was sorted by log2 fold change in macrophages from tumor compared to those from normal lung.

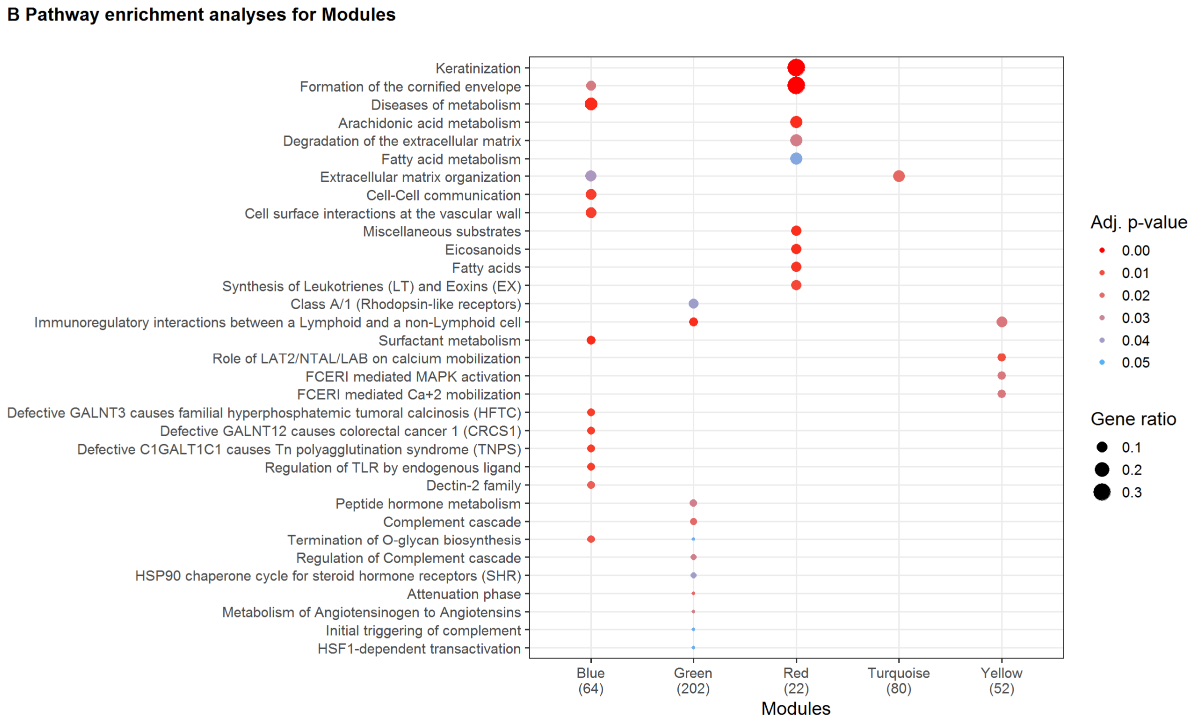

Figure S7. GO enrichment analysis of the module color genes, Legend: Reactome pathway enrichment analysis was performed on the module color genes defined in the WGCNA network clustering analysis. The list of genes in each module was analyzed irrespective of the cell type from which the dataset was generated. The symbol size visualizes visualizes the ratio of genes identified in our data to the genes present in the gene set, the color indicates the adjusted p-value. Enrichment of the Reactome pathway 'Immunoregulatory interactions between a Lymphoid and a non-Lymphoid cell' and 'Class A/1 (Rhodopsin-like receptors)' was observed in the green module.

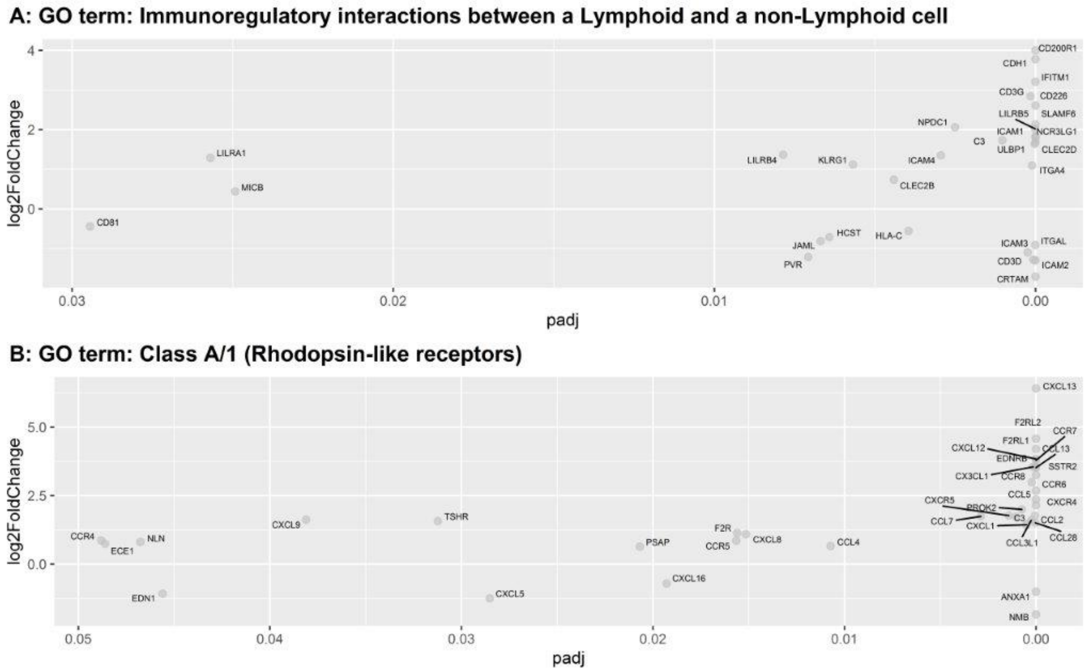

Figure S8. Genes from GO terms related to immunoregulatory interactions between lymphoid and non-lymphoid cells are upregulated in macrophages from tumor compared to normal lung. Legend: Genes in the two Reactome pathways (**A**) 'Immunoregulatory interactions between a Lymphoid and a non-Lymphoid cell' and (**B**) 'Class A/1 (Rhodopsin-like receptors)' that were very significantly up- or downregulated in macrophages from tumor compared to normal lung are shown to visualize highly differentially expressed genes in these two gene sets (high log2 fold change and low adjusted

p-values). Of particular interest are the genes in the upper right corner of both plots, the Ig superfamily member CD200 receptor (CD200R1) (**A**), and the chemokine ligand CXCL13 and its receptor CXCR5 (**B**).

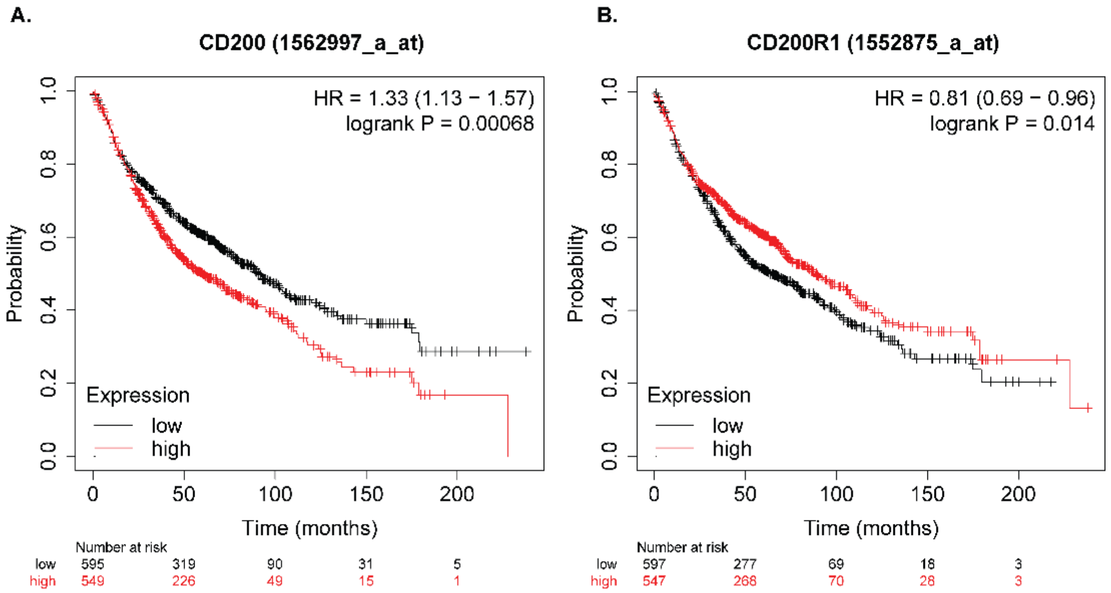

Figure S9. High expression of CD200 in lung cancer tissue samples is associated with decreased survival, Legend: Using the web based Kaplan-Meier plotter, KMplot.com, accessing data on survival data for 54k genes in a large cohort of patients with NSCLC (n = 3452), we looked at the association of expression of CD200 and CD200R in tissue samples on lung cancer survival. While high CD200 (Affymetrix ID: 1562997_a_at) expression was associated with decreased survival (Hazard ratio = 1.33), high CD200R1 (Affymetrix ID: 1552875_a_at) expression was associated with increased survival.
